# Sequencing effects of balance and change of direction training on physical fitness in young male and highly trained soccer players

**DOI:** 10.1038/s41598-025-32748-7

**Published:** 2025-12-19

**Authors:** Lobna Aliani, Raouf Hammami, Andrew Sortwell, Walid Selmi, Haithem Rebai, Urs Granacher

**Affiliations:** 1 Higher Institute of Sport and Physical Education of Ksar-Said, University of Manouba, University Campus, Manouba, 2010 Tunisia; 2 Tunisian Research Laboratory ‘Sports Performance Optimization’, National Center of Medicine and Science in Sports (CNMSS), (CNMSS-LR09SEP01), Tunis, Tunisia; 3 School of Education, the University of Notre-Dame Australia, Sydney, Australia; 4 School of Health Sciences, University of Notre Dame Australia, 32 Mouat St, Fremantle, Australia; 5 Research Centre in Sports, Health and Human Development, University of Beira Interior, Covilhã, Portugal; 6 Research Laboratory (LR23JS01) ”Sport Performance, Health & Society”, Tunis, Tunisia; 7 Department of Sport and Sport Science, Exercise and Human Movement Science, University of Freiburg, Sandfangweg 4, 79102 Freiburg, Germany

**Keywords:** Agility, Stability, Sport specific performance, Training prescription, Football, Physiology, Health care

## Abstract

The capacity to quickly change directions is a critical success factor in soccer. Accordingly, change-of-direction training (CODT) should be part of soccer training. Whether there is a sequencing effect of CODT with other training modalities is currently unresolved. Thus, the study objective was to examine the sequencing effects of balance training (BT) and CODT on selected measures of physical fitness and soccer-specific performance in highly-trained young soccer players. Thirty-seven highly-trained male pubertal soccer players aged 12–13 years (Tier 3) exercised for eight weeks with two weekly CODT (forward, backward and lateral drills) or BT (e.g., bi- and unilateral exercises on unstable surfaces) sessions included in regular soccer training sessions. While experimental group 1 (*n* = 18) performed four weeks of BT followed by four weeks of CODT, group 2 (*n* = 19) followed the opposite sequencing scheme (CODT before BT). BT or CODT lasted 20 min per session and replaced parts of the soccer-specific training, including technical, tactical drills and small-sided games. Pre and post-training, tests were conducted for the assessment of static, dynamic balance (i.e., center of pressure surface area, velocity on firm, foam surfaces), linear sprint speed (i.e., 5-m, 10-m, 30-m) and change-of-direction (COD) ability (i.e., 15-m COD ability test) with and without the ball. Vertical and horizontal jump performances were tested using the countermovement and the standing long jump tests. Once statistical assumptions were met, a two-way repeated-measures analysis of variance (ANOVA) was computed with the factors ‘group’ (BT before CODT vs. CODT before BT) as the between-subject factor and ‘time’ (pre-test vs. post-test) as the within-subject factor. Findings showed significant group-by-time interactions for all proxies of static (*d* = 0.45–1.12; all *p* < 0.01) and dynamic balance (*d* = 0.40 − 0.27; all *p* < 0.03), COD with (*d* = 0.64; *p* < 0.02) and without the ball (*d* = 0.24; *p* < 0.04), horizontal and vertical jump performances (*d* = 1.89–2.94; all *p* < 0.001) and linear sprints (*d* = 0.29–0.73; all *p* < 0.04). Post-hoc tests indicated significant pre-post changes for all tested variables for the group that performed BT before CODT (*d* = 0.28–1.97; all *p* < 0.05) and to a lesser extent for the opposite sequencing scheme (*d* = 0.10–0.28; all *p* < 0.05). A mesocycle commencing with BT prior to CODT appears to have a preconditioning effect, resulting in better outcomes in balance, speed, and jump performances in highly-trained young soccer players. Strength and conditioning professionals working with young male soccer players may apply a block of BT before CODT to enhance their players’ performance.

## Introduction

Linear sprint and change-of-direction (COD) speed, dynamic balance, and jump performance represent important physical fitness qualities related to soccer-specific performance. For instance, Benounis, et al^1^. examined associations between linear sprint (5-m, 20-m split times), COD speed (COD-15 m with and without the ball), and soccer-specific performance (Loughborough soccer passing test, LSPT) in 14-year-old male soccer players. These authors reported moderate-to-high correlations (*r* = 0.71–0.75; *p*< 0.01) between COD speed and soccer-specific performance, emphasizing the need to test and train COD speed in youth soccer. Similarly, Viran, et al^2^. reported significant associations (*r* = 0.23–0.44; *p* < 0.05) between dynamic balance (Y-balance test) and LSPT performance in 15-year-old male players, which further highlights the link between specific physical fitness qualities (COD, balance) and soccer-specific performance in youth players. Collectively, these correlational studies underline that dynamic balance is a significant determinant of COD and soccer-specific performance in youth soccer players, justifying its integration into youth soccer players’ testing and training programs.

Building on these associations, Sariati, et al^3^. investigated the effects of an eight-week COD training (CODT) program combined with soccer-specific training compared to soccer-specific training alone on physical fitness in pre- and post-pubertal male players. The combined program produced greater improvements in balance, horizontal jump, and COD performances (all *p* < 0.04, d = 0.51–1.51) compared with soccer-specific training (*p* < 0.01, d = 0.05–0.20,). Likewise, researchers from previous studies have shown that BT has the potential to enhance not only measures of static and dynamic balance but also sport-specific performance^4,5^. For example, Mitrousis, et al^6^. found that BT combined with soccer training significantly improved balance and soccer-specific technical skills such as passing, ball dribbling, and shooting compared to soccer training only.

Taken together, these research findings raise the question of whether the sequencing scheme of BT and CODT has an impact on training adaptations. In a previous study, Hammami, et al^7^. compared the effects of four weeks of BT followed by plyometric training with the reverse order in male players aged 13–15 years and observed greater improvements in balance, leg stiffness, and horizontal jump performance when BT preceded plyometric training. Of note, BT has the potential to enhance afferent sensory input and efferent motor output^8^. Thereby, it may also prime players for subsequent high-intensity COD movements by facilitating CODT training effects. In contrast, sequencing CODT first may induce fatigue and thereby limit training adaptations. Despite the widespread use of BT and CODT, little is known on the impact of the training sequencing scheme on neuromuscular responses and long-term training adaptations.

Given the importance of both training modalities for performance enhancement in soccer, we aimed to examine the sequencing effects of BT and CODT on static, dynamic balance, horizontal and vertical jump performances, linear sprint and COD speed in male, pubertal and highly-trained soccer players. With reference to the relevant literature^7,9^, we hypothesized that the sequencing scheme of four weeks BT followed by four weeks CODT may induce larger physical fitness improvements compared with the opposite sequencing scheme in young soccer players. Addressing this gap in the literature may inform evidence-based training prescriptions to maximize developmental and performance outcomes during critical phases of youth athletic development.

### Study design

This study is a randomized controlled trial that set out to examine the sequencing effects of BT and CODT on physical fitness in male pubertal and highly-trained soccer players. The independent variables are the different training modalities (BT, CODT) and how they interact according to the sequencing scheme. In addition, the main dependent variables comprise soccer-related physical fitness tests (e.g., balance, jump, COD speed tests) that were assessed pre and post the 8-week intervention program. Participants were randomly assigned to the sequencing scheme BT before CODT or CODT before BT. Both training groups participated in an 8-week training program with two sessions per week in addition to the regular soccer training that focused on the promotion of soccer-specific technical drills. Training volumes were similar between groups.

A passive control group was not included in this study because, ethically, it is not feasible to refrain athletes from practicing their sport. In addition, there is evidence that both training regimes (i.e., BT, CODT) proved to be effective previously when applied as single-mode interventions^3,6^. Pre and post-training, physical fitness tests were applied, including static and dynamic balance, vertical and horizontal jump performances, linear sprint speed and COD ability. A dynamic warm-up program was scheduled before each main training session, involving low-intensity jogging, dynamic stretching, low-intensity forward, sideway, and backward running, acceleration runs, submaximal vertical and horizontal jumping.

## Methods

### Participants

The sample size estimation was computed using G*Power (version 3.1.6). Based on findings from a related study^7,10^ examining the effects of combined BT and agility versus combined agility and plyometric training on 30-m linear sprint speed in young male soccer players (Tier 3), an a priori power analysis with a type I error of 0.01 and 90% statistical power, and an Cohen’s *f* = 0.345 was computed. The analysis indicated that overall, 36 participants would be needed to observe a significant interaction effect for the 30-m linear sprint speed test. In order to avoid falling below the estimated sample size due to players’ sickness or injuries, 37 soccer players aged 12–13 years were recruited for this study. All participants were part of a structured soccer academy program and engaged in regular team-based technical, tactical training. However, to minimize confounding effects, only players without prior strength training experience were included. During the study period, players were excluded who additionally performed strength training outside of the team training. By applying these strict exclusion criteria, we wanted to avoid having another interacting factor that could stimulate or mitigate training adaptations.

In addition, peak height velocity (PHV) is a marker of biological maturation that strongly influences neuromuscular and physical fitness development such as balance, speed, muscle strength and power. Accordingly, it is important to establish the maturational status when doing research with the general youth population or young athletes.

According to McKay, et al.^11^, the training and performance calibre of the participating soccer players can be classified as Tier 3. In other words, the participating players were highly-trained and competed regularly at a regional or national level, typically within an organized club or academy structure. All participants and their legal guardians were fully informed about the nature, purpose, and potential risks of the study before participation. Written informed consent was obtained from both the participants and their legal guardians prior to inclusion in the study. Consent for publication of anonymized data and any potentially identifying information/images in an online open-access publication was also obtained from all participants and their legal guardians. This study was conducted following the latest version of the Declaration of Helsinki and the protocol was approved by the Local Ethics Committee of the National Centre of Medicine and Science of Sports of Tunis (CNMSS-LR09SEP01) before the commencement of the study.

### Procedures

Two weeks before the start of the study, a familiarization session was scheduled to allow the participating athletes to become acquainted with the applied tests and exercises, thereby minimizing potential learning effects during actual testing. BT was implemented two times per week for eight weeks, with each session lasting approximately 20–25 min. Exercises were performed mostly barefoot to enhance somatosensory feedback. Programming during BT included progression from stable to unstable surfaces over the course of the intervention. Each session comprised five exercises, 1–3 sets per exercise, and each exercise was performed for 30–40 s with a 30 s rest between sets.

The assessment of soccer-related physical fitness included a COD ability test (15-m dribbling test with and without a ball), linear sprint speed tests over 5-m, 10-m, and 30-m, static and dynamic balance tests on firm and foam surfaces as well as vertical and horizontal jump tests. The same test sequence was applied during pre and post-tests. Participants received standardized instructions for the technically sound performance of the physical fitness tests.

To ensure coherence in performing the jump, balance, and speed tests, all participants received standardized instructions and demonstrations prior to each assessment, followed by a familiarization session to practice the required movements. Testing procedures were carried out under the supervision of experienced examiners who carefully monitored the execution of each trial, and only data from technically sound trials were used for further analysis. Moreover, all assessments were conducted at the same time of day, on the same surface using identical equipment to minimize data variability.

### Anthropometric and body composition

Anthropometric and body composition assessments were conducted during the familiarization sessions. Body height and mass were measured using a wall-mounted stadiometer and an electronic scale. The dominant leg was identified as the preferred leg used to kick a soccer ball. Skinfold thickness was assessed using Harpenden skinfold calipers (Baty International, West Sussex, England), with measurements taken on the right side of the body. A trained investigator performed triplicate measurements at four sites: biceps, triceps, subscapular, and supra-iliac. Finally, the sum of these skinfolds was calculated for body composition analysis. Body measurements were conducted according to Deurenberg, et al^12^. who reported similar prediction errors between adults and young adolescents. Deurenberg’s prediction equation is as follows: body fat percentage = 222.23 + 26.56 × log (Biceps, triceps, subscapular, and suprailiac). Biological maturity was estimated non-invasively using chronological age, standing height, and sitting height to predict age at peak height velocity (PHV), as previously described by Moore, et al.^13^. This method has been validated for adolescent males and has a standard error of estimate of 0.542 years. No differences in PHV data were observed at baseline (Table 1).

**Table 1:** Anthropometric and body composition characteristics of the pubertal, male and highly-trained soccer players at baseline.

Variables	Sequencing scheme		*p*-value
	BT before CODT group (*n* = 18)	CODT before BT group (*n* = 19)	
Age (years)	13.5 ± 0.3	13.5 ± 0.4	0.60
Body height (cm)	169.6 ± 0.9	170.0 ± 0.8	0.20
Body mass (kg)	59.5 ± 2.1	60.6 ± 1.7	0.10
%BF	11.9 ± 2.6	11.5 ± 2.1	0.48
TE	2.1 ± 0.3	2.1 ± 0.3	0.60
PHV	0.7 ± 0.3	0.1 ± 2.2	0.40
APHV (years)	12.8 ± 0.4	12.3 ± 0.1	0.30

Note: Values as means ± standard deviations; BT: balance training; CODT: change-of-direction training; %BF: body fat percent; PHV: peak height velocity; APHV: age at peak height velocity; TE: training experience.

### Physical fitness tests

All physical fitness tests and warm-up routines were supervised and conducted by certified strength and conditioning specialists with a minimum of five years of experience working with youth soccer players. The same personnel were responsible for delivering standardized warm-up protocols before each test session to ensure consistency and to minimize injury risk. All test administrators received training in the specific test protocols used in this study. Familiarization sessions were scheduled to ensure correct execution and reduce learning effects. The pre-testing warm-up included 5-min jogging at self-selected comfortable pace followed by 5-min of dynamic stretching (i.e., hip flexion/extension, hip abduction/adduction, butt kicks). The fitness tests were scheduled at the same time of day (i.e., 3 pm) during pre and post-tests.

#### Static and dynamic balance

**Fig. 1:**
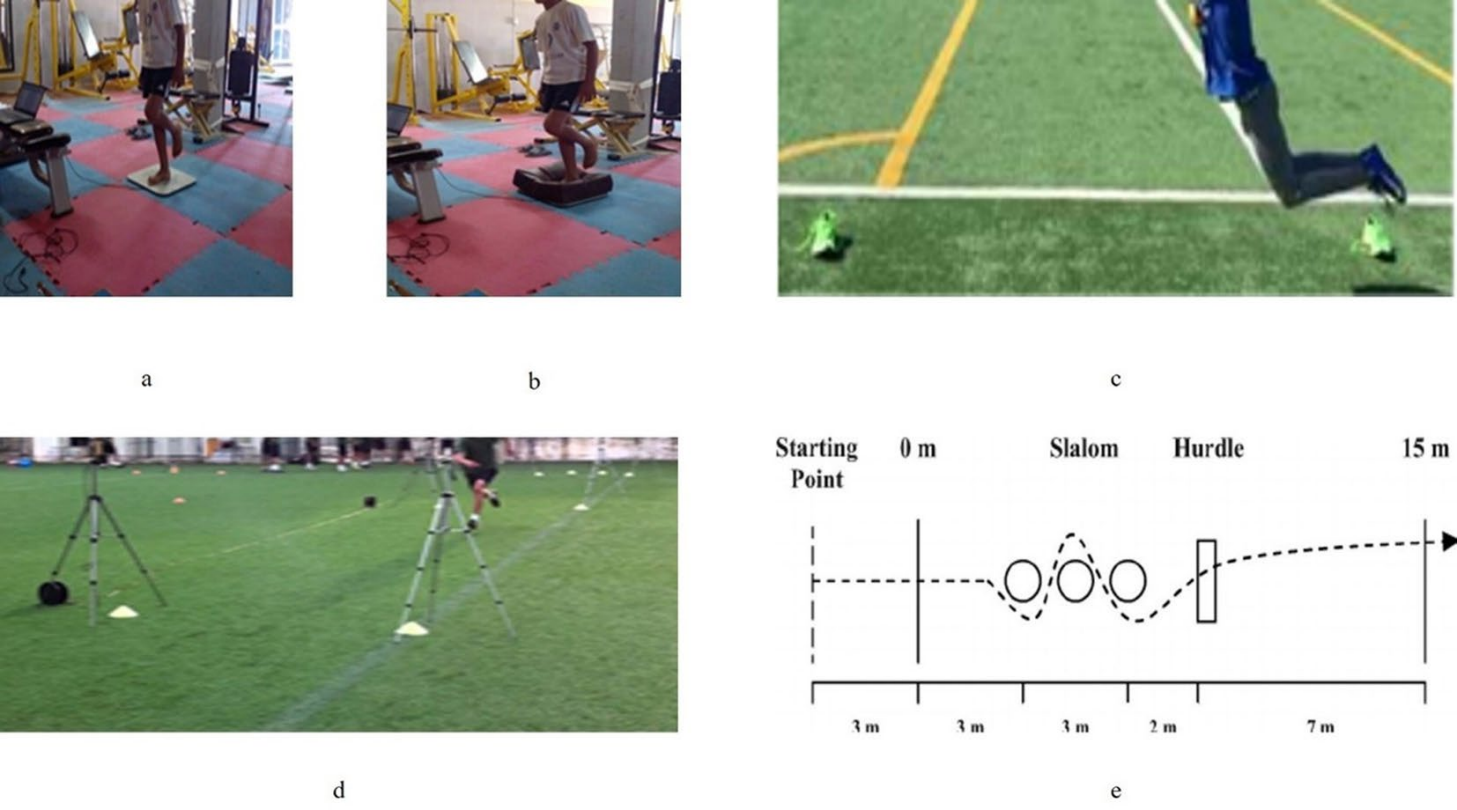
Illustrations of the applied fitness tests. **a**: static balance test; **b**: dynamic balance test; **c** standing long jump test; **d**: linear sprint speed test; **e**: change of direction ability tests with and without the ball.

Static balance was evaluated in a unilateral stance with eyes open on firm surface using a force plate with four strain gauges (PostureWin©, Techno Concept ^®^, Cereste, France). Center of pressure displacements (COP) was assessed for a duration of 30 s at a sampling rate of 40 Hz while standing on the dominant leg. During testing, participants were asked to look straight ahead at a cross, placed at eye level on a nearby wall (2 m distance) (Fig. 1, a). As dependent variables, two center of pressor (COP) sway parameters were analyzed (center of the pressor surface area [COP SA] in mm² and velocity [COP V] in mm/ms)^14^. The lower the COP displacements, the better the balance performance^15^. Dynamic balance performance was tested using the same posturographic platform in a single leg stance (dominant leg) for 30 s and with eyes opened but on an unstable dome-shaped surface (i.e., balance dome) (Fig. 1, b). With our own data, test-retest reliability was good with ICC = 0.80–0.84 and SEM = 4.32–6.66%.

#### Vertical and horizontal jump performance

The countermovement jump (CMJ) test was performed using an Ergojump^®^ system (Globus Italia, Codognè, Italy). The Ergojump system consists of a contact mat to estimate jump height using flight time derived from ground contact time and take-off detection. The system provides output for jump height (in cm), contact time, and flight time at a sampling rate of 1000 Hz, as specified by the manufacturer. Each test was separated by a passive recovery period of 5 min. The CMJ involved participants descending from an upright standing position until a knee flexion angle of 90°, followed immediately by an explosive leg and trunk extension. The CMJ was performed with hands on hips to eliminate the effects of arm swing. Participants performed three trials and the trial with the best performance was used for further analysis.

Horizontal jump performance was tested using the standing long jump (SLJ). Participants stood in front of a starting line in upright, erect stance and were instructed to push-off vigorously and jump forward as far as possible. Participants were allowed to perform a countermovement with arm swing. The distance (cm) jumped from the starting line at takeoff to the position of the heel upon landing was measured in centimeters using a metal tape measure (Fig. 1, c). Participants completed three trials, with the highest score being recorded. Our own data indicated excellent test-retest reliability with ICC = 0.94–0.97 and SEM = 0.22–3.48%.

#### Linear sprint speed

The 5-m, 10-m, and 30-m sprint distances were selected to evaluate both, acceleration and maximum sprint speed, which are critical components of match-play performance in youth soccer^16^. The 5-m and 10-m sprints capture short-distance acceleration, which is particularly relevant in small-area play and transitions. The 30-m distance allows for the assessment of top-end sprint speed, which is often reached between 20 and 30 m in adolescent athletes.

Although 20-m sprints are commonly used in youth testing, we decided not to include the 20-m to avoid redundancy and participant fatigue, since performance over 20 m can be reasonably inferred from the 5, 10-m and 30-m split data. Additionally, the selected distances have been validated and widely used in similar populations to record speed development and training adaptations^17^.

Linear sprint times were recorded using electronic photocell timing gates (Brower Timing Systems, Salt Lake City, UT, USA) with an accuracy of 0.001 s. Gates were positioned at the start line, as well as at the 5-m, 10-m, and 30-m marks, each placed approximately 0.4 m above the ground to align with the athletes’ torso height and reduce early triggering by limb movement. Participants began each sprint from a static, two-point standing position, with their front foot placed 0.5 m behind the first timing gate to ensure the start was based on movement initiation, not a false trigger. Players were instructed to sprint maximally through the final gate (Fig. 1, d). The best time from three valid trials was used for analysis, with at least 2 min of passive recovery between efforts. Test-retest reliability was excellent, with ICC = 0.91–0.98 and SEM = 0.15–0.80%.

#### Change-of-direction speed with and without the ball

COD performance was assessed using the 15-m dribbling test with and without a ball, which involves a 180° turn following a linear sprint, emphasizing braking and re-acceleration. This test has been shown to reliably assess COD speed in youth athletes^18^. The 15-m COD speed test was administered following the standardized protocol outlined by Mujika, et al.^18^. Apart from high overall validity^18^, the test has an advantage in terms of ecological validity as it includes generic cues that closely replicate the majority of movement patterns performed in soccer. The players started the test with a 3-m linear acceleration, followed by a 3-m slalom section marked by three aligned sticks placed 1.5 m apart, then cleared a hurdle placed 2 m beyond the third stick (Fig. 1, e). Finally, the players finished the test with a 7-m linear sprint.

Based on our own data, test-retest reliability was excellent with ICC = 0.96 and SEM = 0.18%.

The COD speed test with ball is similar to the COD test without a ball. While performing the test, players had to dribble the ball (circumference of 68–70 cm [27–28 in]; mass 410–450 g). The difference between the two scores, COD test with the ball minus COD test without the ball, represented the time required for ball control techniques and was the third score obtained from the COD ability test, named TECHNINDEX^19^. For each test, players performed three trials (3 min rest between trials), and the best performance was selected for analysis. Data from our own laboratory indicated excellent test-retest reliability with ICC = 0.90 and SEM = 0.46%.

### Training programs

The sequencing scheme BT before CODT (BT-CODT) involved completing four weeks of BT followed by four weeks of CODT. The group that performed the sequence CODT before BT (CODT-BT) completed four weeks of CODT followed by four weeks of BT. Training volume, defined as session duration and weekly training frequency, and training intensity were matched between the two experimental groups to ensure comparability of the overall training load. Both training interventions were conducted on the soccer pitch under similar environmental and contextual conditions.

#### Balance training

The BT program consisted of five exercises, including (1) kneeling on a Swiss ball with eyes opened and closed, (2) bilateral and unilateral stance on an inflated disk performing squat exercises, (3) supine straight leg bridge on a Swiss Ball, (4) lunges performed on a foam surface (balance pad, inflated disk, BOSU ball) while holding dumbbells, and (5) performing bilateral squats with elastic band straps (Theraband, Akron, OH, United States) attached to a bar placed on the shoulders while standing on a foam surface (balance pad, inflated disk, BOSU ball) (Table 2). Each balance exercise during BT lasted 30–40 s and was repeated 1–3 times with 8–12 repetitions and a 30 s rest between sets. Each balance exercise was performed with the objective of maintaining balance while performing the task.

**Table 2:** Design of the balance training program (BT).

Exercises	Session1 Sets × reps	Session 2 Sets × reps	Session 3 Sets × reps	Session 4 Sets × reps	Session 5 Sets × reps	Session 6 Sets × reps	Session 7 Sets × reps	Session 8 Sets × reps
Kneeling on a Swiss ball, progressing from eyes open to eyes closed	1 × 30	2 × 30	1 × 40	2 × 40	1 × 30	3 × 40	3 × 45	1 × 30
Unilateral and bilateral standing balance exercise on inflated disc progressing to squat exercise	1 × 8/leg	2 × 10/leg	2 × 12/leg	2 × 15/leg	1 × 10/leg	3 × 12/leg	3 × 15/leg	1 × 10/leg
Supine straight leg bridge on Swiss ball	1 × 8/leg	2 × 10/leg	2 × 12/leg	2 × 15/leg	1 × 10/leg	3 × 12/leg	3 × 15/leg	1 × 10/leg
Lunge on foam surface progressing to BOSU ball or inflated disc with holding dumbbells	1 × 8/leg	2 × 10/leg	2 × 12/leg	2 × 15/leg	1 × 10/leg	3 × 12/leg	3 × 15/leg	1 × 10/leg
Bilateral squat with elastic straps attached to a bar placed on the shoulders on a foam surface progressing to BOSU ball or inflated disc	1 × 8	2 × 10	2 × 12	2 × 15	1 × 10	1 × 12	1 × 15	1 × 10

**Notes**: Sets: series; reps: repetitions.

#### Change-of-direction speed training

CODT included cutting maneuvers and sprint drills that incorporated planned directional changes at 45°, 90°, and 180°. Progression involved increasing the linear sprint speed approach before entry into the turn, reducing ground contact time, and introducing soccer-specific stimuli. For example, athletes performed sprint and cut drills at 45° to mimic diagonal attacking runs, lateral shuffles with 90° COD to simulate defensive transitions, and deceleration and pivot drills with 180° turns replicating recovery movements. These angles were incorporated into the exercises to ensure the training was ecologically valid, targeting a range of COD tasks.

CODT included ladder drills (forward sprint with high knee flexion, lateral shuffle, hop in and out, quick-feet ladder sprint), cone drills (oblique shuttle runs, agility t-drill, forward t-drill, and backward t-drill) and drills with and without the ball (Table 3). A recovery time of approximately 50 s was allowed between repetitions and 2–3 min between sets.

**Table 3:** Design of the change-of-direction training program (CODT).

Exercises	Figures	Session1 sets × reps/or (s)	Session 2 sets × reps/or (s)	Session 3 sets × reps/or (s)	Session 4 sets × reps/or (s)	Session 5 sets × reps/or (s)	Session 6 sets × reps/or (s)	Session 7 sets × reps/or (s)	Session 8 sets × reps/or (s)
Forward sprints with high knee flexion		1 × 10 (s)	2 × 10 (s)	3 × 10 (s)	3 × 10 (s)				
Lateral high knee flexion, lateral shuffles		1 × 10 (s)	2 × 10 (s)	3 × 10 (s)	3 × 10 (s)				
Forward sprints with high knee flexion, quick-feet ladder sprints						2 × 10 (s)	4 × 10 (s)	5 × 10 (s)	1 × 10 (s)
Oblique shuttle runs: forward 5 m, and then crossover strides 5 m and forward 5 m and then crossover strides 5 m		1 × 4 (reps)	1 × 5 (reps)	2 × 5 (reps)	2 × 6 (reps)	1 × 5 (reps)	3 × 5 (reps)	3 × 6 (reps)	1 × 4 (reps)
T-drills: on the start command, athletes accelerated to their right and back 5 m, ran forward and back 5 m, ran backward and back 5 m and then to the left and back 5 m at full speed		1 × 2 (reps)	1 × 5 (reps)	1 × 6 (reps)	1 × 7 (reps)	1 × 2 (reps)	2 × 5 (reps)	2 × 6 (reps)	1 × 2 (reps)
Forward and backward t-drills: sprint forward/backward 5 m and move laterally 5 m upon command		1 × 6 (reps)	2 × 8 (reps)	2 × 10 (reps)	2 × 12 (reps)	1 × 10 (reps)	3 × 8 (reps)	3 × 10 (reps)	1 × 8 (reps)
Square cone drills: sprint forward 5 m, shuffle laterally 5 m, sprint backward 5 m and shuffle laterally to the starting point		1 × 4 (reps)	2 × 5 (reps)	2 × 6 (reps)	2 × 7 (reps)	1 × 5 (reps)	1 × 6 (reps)	1 × 7 (reps)	1 × 2 (reps)
x-square cone drills: sprint forward 5 m, sprint back with crossover strides obliquely to the back right corner 5 m, sprint forward 5 m and sprint back with crossover strides obliquely to the back left original corner 5 m		1 × 4 (reps)	2 × 5 (reps)	2 × 6 (reps)	2 × 7 (reps)	1 × 5 (reps)	1 × 6 (reps)	1 × 7 (reps)	1 × 2 (reps)
Two-metre acceleration with the ball, then slalom dribbling with the ball between 3 cones, and finally pass the ball. After that, a 5 m acceleration without the ball is performed over a 10 m distance.		1 × 3 (reps)	1 × 4 (reps)	2 × 3 (reps)	2 × 4 (reps)	1 × 5 (reps)	3 × 5 (reps)	3 × 6 (reps)	1 × 4 (reps)

Note: (s) = seconds; reps: repetitions.

### Statistical analyses

Before computing the statistical analyses, data were checked for normality using the Shapiro–Wilk test and for homogeneity of variances using the Levene’s test, in accordance with the assumptions required for parametric testing. Once assumptions were met, a two-way repeated-measures analysis of variance (ANOVA) was computed with the factors ‘group’ (BT before CODT vs. CODT before BT) as the between-subject factor and ‘time’ (pre-test vs. post-test) as the within-subject factor. If group-by-time interactions reached the significance level, group-specific and Bonferroni-corrected post-hoc tests (i.e., paired sample t-tests) were calculated to identify group-specific pre-post changes. Effect sizes were reported as partial eta squared (η²ₚ) for main and interaction effects derived from ANOVA, and converted into Cohen’s d. The within-group pre-post effect sizes were calculated as: d = M1-M2/SDp (pooled standard deviations).

Cohen’s d was classified as trivial (0.00 ≤ d ≤ 0.19), small (0.20 ≤ d ≤ 0.49), medium (0.50 ≤ d ≤ 0.79), and large (d ≥ 0.80)^20^. Cronbach’s model of intra-class correlation coefficients (ICCs 3.1) and standard error of measurements (SEM) were computed according to the methods as introduced by Hopkins, et al.^20^. At baseline, two trials were used for each variable to analyze intra-session reliability^21^. Test-retest reliability was assessed using data from all participants. ICCs were interpreted as follows: < 0.40 = poor, 0.40–0.70 = fair, 0.70–0.90 = good, and > 0.90 = excellent^20^. The SEM was calculated by dividing the SD of the difference between scores by √2. To verify the equivalence of groups at baseline, independent sample t-tests were calculated for all continuous variables (e.g., age, height, body mass, CMJ height, sprint time, COD performance). Statistical significance was set at *p* < 0.05, and all analyses were performed using SPSS v27.

## Results

No test or training related injuries occurred during the study period. Training adherence amounted to 96% for the BT-CODT group and 98% for the CODT – BT group. No significant between-group baseline differences were observed for all analyzed variables (Table 1).

### Static and dynamic balance 

**Fig. 2:**
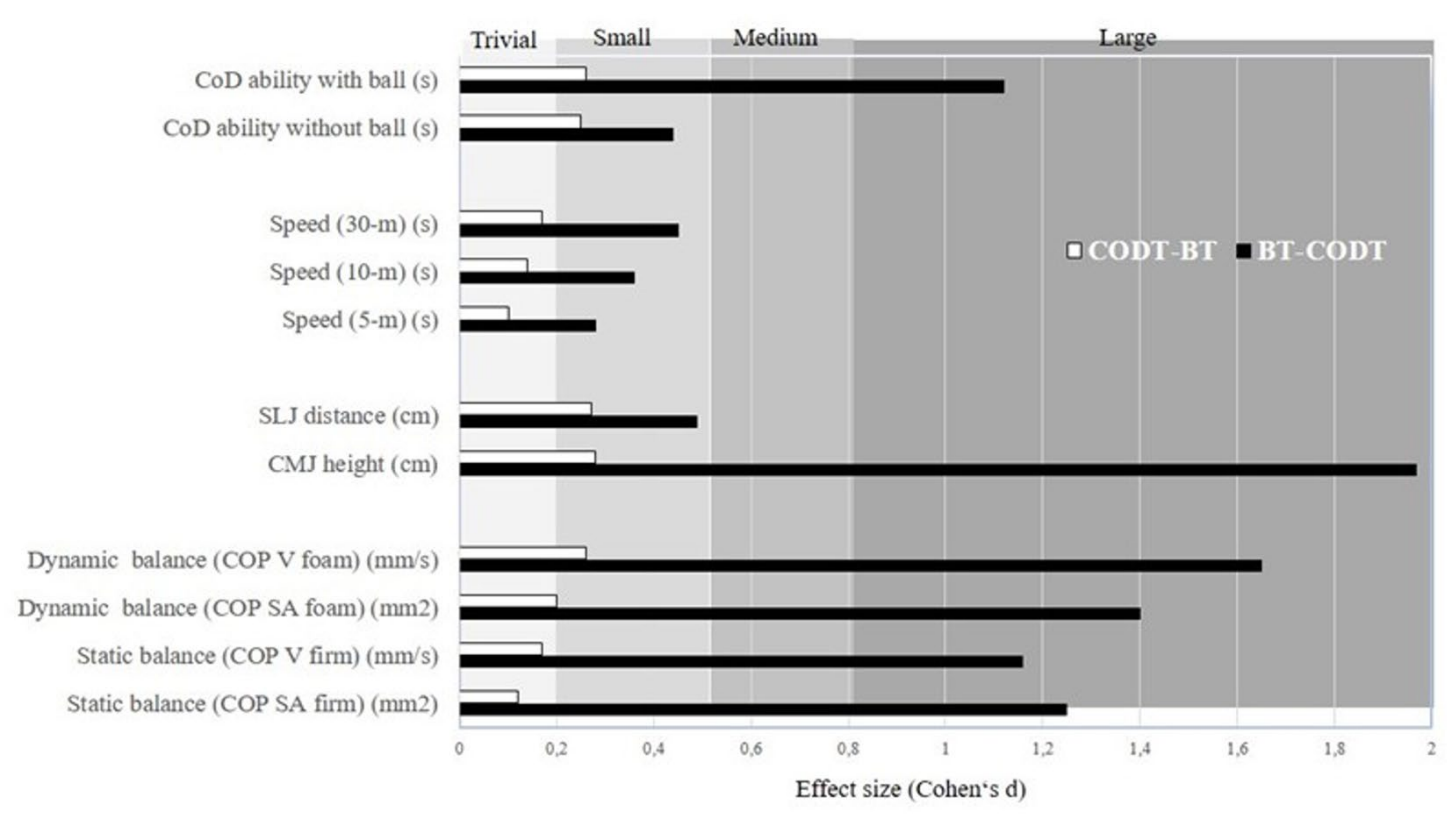
Summary of the study findings illustrated as effect sizes from group-specific pre-post changes for each measured variable based on significant group-by-time interactions.

For static balance, findings indicated significant group-by-time interactions for COP SA firm and COP V firm (*d* = 0.45–1.12, all *p* < 0.01) (Table 3). Post-hoc analyses showed a significant pre-post COP SA firm and COP V firm performance improvement for the BT-CODT group (∆32.9–30.1%; *p* < 0.0001; *d* = 1.25–1.16) and a smaller improvement for the CODT-BT group (∆7.8–17.8%; *p* < 0.001; *d* = 0.12–0.17) (Fig. 2; Table 4).

**Table 4:** Group-specific means and standard deviations for all outcome measures before (pre) and after (post) the intervention period.

	Sequencing scheme BT before CODT group (*n* = 18)				∆ %	CODT before BT group (*n* = 19)				∆ %	ANOVA			
	pre test		post test			pre test		post test			*p*-value (d)			
	M	SD	M	SD		M	SD	M	SD		Time	Group		Group × Time
Static balance performance														
*COP SA firm (mm* ^*2*^ *)*	966.2	85.2	726.9	79.9	32.9	932.6	68.3	864.5	73.8	7.8	<**0.001 (3.25)**	<**0.02** **(0.33)**		<0.002* **(1.12)**
*COP V firm (mm/s)*	39.3	4.2	30.2	3.2	30.1	38.1	5.6	32.5	2.8	17.1	<**0.001 (2.90**	<**0.01** **(0.53)**		<0.01* **(0.45)**
Dynamic balance performance														
*COP SA foam (mm* ^*2*^ *)*	1323.5	159.1	911	109.6	45.2	1285.3	102.8	1099.2	103.7	16.9	<**0.001 (2.99)**	<**0.01** **(0.22)**		<0.02* **(0.40)**
*COP V foam (mm/s)*	46.4	5.8	32.5	4.5	42.6	45	5.6	37.5	4.1	20	<**0.001 (3.15)**	**0.03** **(0.18)**		<0.03* **(0.27)**
Vertical and horizontal jump performances														
*CMJ-height (cm)*	20.9	1.2	25.7	1.4	22.7	20.6	0.9	22.5	1	8.9	<**0.001 (14.37)**	<**0.02** **(0.66)**		<0.001* **(2.94)**
*SLJ distance (cm)*	194.1	7	218.1	5.9	12.3	194.9	5	202.5	5.1	4.3	<**0.001 (12.24)**	<**0.01** **(0.44)**		<0.001 * **(1.89)**
Linear sprint speed														
*5-m (s)*	1.2	0.02	1.1	0.02	7.6	1.2	0.1	1.2	0.03	3.3	<**0.018 (0.84)**	<**0.01** **(0.59)**		<0.229* **(0.29)**
*10-m (s)*	2.2	0.03	2.1	0.02	4.1	2.2	0.03	2.2	0.04	3.6	<**0.001 (0.51)**	<**0.02** **(0.36)**		<0.046* **(0.70)**
*30-m (s)*	5.3	0.1	5.1	0.1	3.6	5.3	0.1	5.2	0.1	1.5	<**0.001 (6.47)**	<**0.02** **(0.25)**		<0.037 * (0.73)
Change-of-direction ability														
*COD ability without ball (s)*	5.1	0.2	4.5	0.2	13.8	5.1	0.2	4.7	0.2	7.5	<**0.001 (5.63)**	**0.02** **(0.42)**		<0.0486* **(0.24)**
*COD ability with ball (s)*	6.8	0.2	6.3	0.2	6.2	6.8	0.3	6.3	0.3	7.2	<**0.001 (13.33)**	**0.03** **(0.06)**		<0.02* **(0.64)**

Notes: Pre- and post-training values (mean ± SD) for all physical fitness measures separated for the two experimental groups. Statistically significant group-by-time interaction effects are marked with an asterisk ().* Landscape orientation was applied to ensure clear presentation of multiple performance metrics across groups and time points. Values as means ± standard deviations; BT: balance training; CODT: change-of-direction training; SLJ: standing long jump; CMJ-height: counter-movement jump height; COD ability: change-of-direction speed; COP SA: center of pressure surface area; COP V: center of pressure velocity; d = Cohen’s d.

In terms of dynamic balance, results indicated a significant group-by-time interaction for both COP SA foam and COP V foam (*d* = 0.27–0.40, all *p* < 0.001). Post-hoc analyses demonstrated a significant performance enhancement from pre-to-post for the BT- CODT group (∆42.6–45.2%; all *p* < 0.001; *d* = 1.40–1.65) and a smaller but still significant improvement for the CODT-BT group (∆16.9–20%; all *p* < 0.001; *d* = 0.20–0.26) (Fig. 2; Table 4).

### Vertical and horizontal jump performances

For CMJ-height, a significant group-by-time interaction (*d* = 2.94, *p* < 0.001) was observed. Post-hoc analyses demonstrated large CMJ improvements for the BT-CODT group (∆22.7%; *p* < 0.001; *d* = 1.97) and a small improvement for the CODT-BT group (∆8.9%; *p* < 0.001; *d* = 0.28) (Fig. 2; Table 4).

For the SLJ test, a significant group-by-time interaction (*d* = 1.89, *p* < 0.001) was noted. Post-hoc analyses indicated a moderate pre-post improvement for the BT- CODT group (∆12.3%; *p* < 0.001; *d* = 0.49) and a small improvement for the CODT-BT group (∆4.3%; *p* < 0.001; *d* = 0.27) (Fig. 2; Table 4).

### Linear sprint speed

For linear sprint speed, significant group-by-time interactions (*d* = 0.29–0.73, all *p* < 0.001) were observed. Post-hoc analyses indicated small improvements for the BT- CODT group (∆3.6–7.6%; all *p* < 0.001; *d* = 0.28–0.36) and trivial improvements for the CODT- BT group (∆1.5–3.6%; all *p* < 0.001; *d* = 0.10–0.14) (Fig. 2; Table 4).

### Change-of-direction speed

For the COD speed tests with and without the ball, significant group-by-time interactions (*d* = 0.24–0.64, *p* < 0.02) were found. Post-hoc analyses indicated small improvements for the BT-CODT group (∆6.2–13.8%; all *p* < 0.05; *d* = 0.44–1.12) and for the BT-CODT group (∆7.2–7.5%; all *p* < 0.001; *d* = 0.25–0.26) (Fig. 2; Table 4).

## Discussion

This study examined the sequencing effects of BT and CODT within an eight-week training program on selected measures of physical fitness in pubertal, male and highly-trained soccer players (Tier 3). The results indicated that the sequencing scheme BT before CODT was more effective than CODT before BT in improving proxies of balance, vertical and horizontal jump performances, linear sprint and COD speed. These results support the study’s hypothesis and, to the best of our knowledge, represent the first evidence demonstrating the advantage of performing BT prior to CODT in pubertal male soccer players.

The findings of this study are in agreement with the recommendations of Behm, et al.^22^, who postulated in their review article that BT should be conducted prior to strength training in youth to enhance training adaptations. This recommendation is grounded in the notion that children’s balance and coordination are still developing, making BT crucial for achieving optimal performance and reducing the risk of sports-related injuries^23,24^. Supporting this perspective, Hammami, et al^7^. proposed that BT may serve as a preconditioning stimulus, particularly enhancing neuromuscular and balance performance in young athletes.

Although previous studies have examined the combined effects of BT and CODT in trained young male soccer players^10,25^, no research to date has specifically investigated the sequencing effects of these training modalities applied for a four week mesocycle BT followed by four weeks CODT and vice versa. Of note, Makhlouf, et al^10^. conducted a similar study with 12- to 15-year-old (Tier 3) soccer players over an eight week training period, where participants performed a combination of CODT, plyometric, and BT exercises within the same training sessions but without a specific training modality sequence. Findings from this study showed that the combined BT and CODT program produced similar effects on measures of physical fitness compared to CODT alone^10^. In contrast, researchers from another study with similar design^25^ reported that a six-week combined CODT and BT program resulted in significantly greater improvements in dynamic balance, linear sprint and COD speed, and drop jump performance compared to a group that underwent only CODT^25^. Further support comes from a narrative review by Gebel, et al.^4^. These authors concluded that BT has the potential to elicit improvements in specific components of physical fitness among youth and young adults, including balance, muscular strength and power, and COD ability. Collectively, the scientific literature and the findings from other researchers align with the results of the current study, suggesting that the sequencing scheme BT before CODT results in larger performance improvements compared with the opposite sequencing scheme in young soccer players.

Even though the literature supports the effectiveness of CODT in youth athletes^26^, particularly for enhancing muscular power^27^, balance^3^, linear sprint speed, and COD ability^28^, the present findings suggest that performing BT before CODT even results in larger improvements compared with the application of CODT alone. There are several possible explanations for our findings. For instance, BT may enhance postural control, thereby preparing individuals for subsequent CODT exercises^29^. COD performance affords high balance demands. The successful integration and processing of somatosensory information is required during the performance of COD tasks to maintain balance in highly dynamic situations^10^.

Large postural sway, either when stationary or during dynamic movement, can project the center of mass beyond an individual’s base of support, resulting in loss of balance and an inability to maintain an upright, erect posture^30^. Adequate postural control is needed to successfully perform in soccer. For instance, maintaining balance during a tackle affords to stabilize the body while changing directions rapidly. Moreover, performance of controlled landings after vertical jumps requires fine-tuned control of the center of mass over the relatively small base of support (feet). There is evidence that young soccer players with enhanced postural control can better maintain stability during these unpredictable and highly-demanding scenarios, especially if opponents are involved^31^. Given that high-speed COD performances impose frequent perturbations of the postural control system, the ability to effectively maintain static and dynamic balance (metastability) can positively impact on athletic (i.e., soccer) performance^32^.

Previously, researchers have demonstrated that BT can have a preconditioning effect, enhancing performance in subsequent blocks of plyometric or strength training, particularly in youth^7^ and adult^9^ populations. The BT – CODT sequencing scheme may help facilitate a smoother transition into sport-specific drills (i.e., transmutation) by better preparing the body to handle increased mechanical loads and intensities. One key physiological mechanism that may explain the superiority of the training sequence BT before CODT is enhanced neuromuscular activation and proprioceptive priming^4,25^. BT activates joint stabilizers and improves sensorimotor feedback, which may help prepare the central nervous system for higher-intensity efforts that require precise coordination, such as sprinting and jumping. This so-called “neuromuscular readiness” can improve muscle synergistic activation during the performance of subsequent COD tasks.

In contrast, CODT before BT may induce acute neuromuscular fatigue due to high training intensities, reducing the effectiveness of the subsequent BT exercises. Given that balance adaptations are sensitive to central and peripheral fatigue of the nervous system^31^, neuromuscular fatigue may attenuate gains in postural control during BT^24^. Furthermore, the group that engaged in CODT during the initial four weeks may have achieved adaptations in sprint, jumping, and COD performances that could have partially diminished (detraining) during the subsequent four weeks of BT. This potential detraining effect might partly explain different training adaptations observed in the two intervention groups.

From a biomechanical perspective, the sequencing scheme BT before CODT may improve postural control through improved lower limb joint alignment, which are important pre-requisites for effective force production and transfer during COD task performance. Improved control over the body’s center of mass during BT may lead to more efficient movement control during sprints, jumps, and directional changes. Conversely, if high-intensity CODT is performed before BT, players may experience altered movement biomechanics due to neuromuscular fatigue or suboptimal muscle activation patterns, potentially limiting balance adaptations and diminishing chronic training adaptations^33^. Hence, performing the BT-CODT sequencing scheme may improve chronic training adaptations through enhanced movement quality.

While the current findings highlight the benefits of BT performed prior to CODT, several potential confounding variables should be acknowledged. First, although training loads were standardized across groups, we did not directly monitor or control changes in lower-limb strength or power over the intervention period. Given the age and developmental stage of the study participants, natural strength gains may have occurred due to maturation-related neuromuscular adaptations, independent of the intervention. This is particularly relevant in the context of the law of diminishing returns, as athletes with lower initial fitness levels often experience more rapid improvements than their more developed peers. Yet, growth and maturation related strength gains would have occurred in both groups. Nevertheless, future studies should incorporate direct strength testing or include strength as a covariate in the analysis. Second, while the Ergojump^®^ system and photocell timing gates are widely used and validated in field testing, they are less sensitive than laboratory-grade force platforms or 3D motion capture systems, especially when assessing subtle biomechanical changes or postural sway. This may limit preciseness of our performance measures but at the same time follows an ecologically valid approach. Lastly, growth and biological maturation, though estimated using PHV, may still act as a residual confounder, as small inter-individual differences in growth rates or hormonal profiles can influence neuromuscular control and adaptation.

Furthermore, although age at PHV (APHV) was estimated, it was not included as a covariate in the statistical analyses. Given the well-established influence of biological maturation on neuromuscular performance in youth athletes—particularly around the PHV period—this may have confounded the interpretation of training effects. Future research should consider including APHV as a covariate or stratifying participants based on the maturity status (e.g., early, on-time, or late maturers) to better account for maturation-related differences.

Recent research findings, such as those by Padrón-Cabo, et al^34^. reported significant detraining effects on sprint and jump performance in circa-PHV soccer players following short-term training cessation. These authors suggested that detraining effects may occur in this population of circa-PHV athletes. Additionally, the absence of a passive control group limits findings from this study in as much as we cannot isolate training-induced changes from those due to natural growth and maturation. Finally, we acknowledge that we did not include objective external load measures, such as GPS data, which is a limitation particularly with regards to the workload achieved during soccer-specific training and CODT. These aspects should be addressed in future research to strengthen the interpretation of training effects in youth pubertal soccer players.

## Conclusions

Findings from this study indicate that BT performed prior to CODT is more effective than the reverse sequencing scheme for improving static and dynamic balance, vertical and horizontal jump performances, linear sprint and COD speed in pubertal, male and highly-trained soccer players (Tier 3).

Coaches as well as strength and conditioning professionals working with male pubertal soccer players are advised to incorporate at least four weeks of BT starting with simple, stationary tasks and advancing to dynamic, sport-specific balance tasks prior to introducing CODT. This sequencing scheme may enhance neuromuscular readiness, coordination, and training responsiveness, leading to greater overall improvements in COD-related performances. Based on the findings of this study, it is recommended that coaches of young male players aged 12–13 years structure their training plans using the sequencing scheme BT before CODT to maximize physical fitness adaptations and to better prepare young players for the multifactorial demands of soccer.

## Data Availability

The datasets used and/or analyzed during the current study are available from the corresponding author upon reasonable request.
